# Clinicopathological Analysis of Neuroendocrine Carcinoma of the Uterine Cervix: A Single-Institution Retrospective Review of 9 Cases

**DOI:** 10.1155/2021/8290659

**Published:** 2021-09-16

**Authors:** Saliha Sağnıç, Özer Birge, Mehmet Sait Bakır, Ceyda Karadag, Tayup Şimşek

**Affiliations:** Akdeniz University, Department of Gynecology Obstetrics, Division of Gynecologic Oncology, Antalya, Turkey

## Abstract

**Aim:**

To evaluate the clinicopathological features affecting the recurrence and survival of 9 cases of neuroendocrine cancer of the cervix.

**Method:**

We retrospectively analyzed 9 cervical neuroendocrine cancer cases identified among 453 cervical cancer patients between 2004 and 2021 at Akdeniz University Gynecological Oncology Outpatient Clinic. Kaplan–Meier survival analysis was used for progression-free survival (PFS) and overall survival (OS). Mathematical functions of mean, standard deviation, median, Min–Max values, and frequencies were used for descriptive statistics. The categorical data were expressed in numbers and percentages (%).

**Results:**

Nine patients with neuroendocrine histological subtype were selected out of 453 patients diagnosed with cervical cancer (1.98%). The average overall survival time of the patients was 26 months. The 5-year survival rate was 53.3%, while the PFS was 62.5%. The most common subtype was small cell neuroendocrine cancer. Tumours were mostly locally advanced at the time of diagnosis. 3 patients' stage was 1b2, while 4 patients were 2b, 1 patient was 3c2r, and 1 patient was 4b. All tumours showed the immunohistochemical staining properties of neuroendocrine cancer. The main treatment modality applied to our patients was surgery + adjuvant CRT. The most used chemotherapeutic agents were cisplatin/carboplatin and etoposide. Recurrence was found in 3 cases, including 5 deaths.

**Conclusion:**

Neuroendocrine tumour of the cervix is a rare subtype with a poor prognosis. Unfortunately, there is not yet a standard treatment protocol due to the limited number of comparative studies of surgery, chemotherapy, and radiotherapy based treatment schemes.

## 1. Introduction

Squamous cell cancer and adenocarcinoma constitute 95% of all diagnosed cervical cancer cases. Neuroendocrine cancer constitutes 1–1.5% of all cervical cancers [1]. These tumours originating from neuroendocrine cells are among the subtypes with a poor prognosis of cervical cancer and have immunohistochemical staining properties similar to endocrine glandular cells. Neuroendocrine tumours of the cervix are categorized as low-grade tumours, which were previously classified as typical carcinoid tumour and atypical carcinoid tumour, intermediate-grade tumours, and high-grade tumours, which were formerly classified as small cell cancer and large cell cancer in 2014 WHO classification system [2]. The standard treatment is not clear due to the rarity of this subtype; therefore, its treatment involves various difficulties. Cancer had an aggressive course due to invasion of the lymphovascular space, lymph nodes, and early hematogenous spread at the time of diagnosis. Therefore, the recurrence-free survival and overall survival of the patients are less compared to other histological types of cervical cancer [1–3].

In the female genital system, neuroendocrine tumours are mostly detected in the ovary, while high-grade neuroendocrine tumours are mostly observed in the cervix [3]. HPV (human papillomavirus), which is an etiologic factor in cervical cancer, has also been observed in cases of neuroendocrine cancer [4, 5]. The main types of HPV that pose a risk for squamous cell cervical cancer have also been identified as a factor in neuroendocrine cancer of the cervix [5]. However, smoking, which is a risk factor in small cell cancer of the lung, has not been identified as a factor in neuroendocrine cancer of the cervix [6]. Clinical presentations of the patients with neuroendocrine tumour of cervix are similar to cervical cancer. However, cancer is more likely to be metastatic at the time of diagnosis compared to other cervical histological types [3].

However, when the literature is reviewed, it should be noted that the studies conducted are generally retrospective studies involving only a small number of patients, and there are no prospective studies [2]. Since most studies investigating the treatment of neuroendocrine tumours are performed on patients with tumours located in organs other than the cervix, mostly the lung or pancreas, the treatment of cervical neuroendocrine tumours requires a multidisciplinary approach [7, 8]. The treatment schemes used in pulmonary neuroendocrine cancer are generally used in the same histological type of cervix cancer because both tumours have similar histological and prognostic features. Following the recommendations of the Society of Gynecologic Oncology (SGO) and Gynecologic Cancer InterGroup (GCIG) [9, 10], treatment schemes for cervical neuroendocrine cancer usually consist of radical hysterectomy followed by adjuvant chemotherapy for the early stages of the disease. Definitive concurrent chemoradiation, neoadjuvant chemotherapy followed by surgery, or chemotherapy alone are described for locally advanced and metastatic diseases [1, 7]. Despite aggressive treatment, neuroendocrine cancer of the cervix still has a poor prognosis. Therefore, it is necessary to analyze the clinicopathological risk factors of patients in detail and develop new treatment schemes or therapeutic agents.

In this study, we aimed to retrospectively review the clinicopathological features of patients with neuroendocrine cancer of cervix affecting recurrence and survival, including age, body mass index, smoking, menopause status, HPV status and type, immunohistochemical staining patterns, gravida, parity, abortion, medical history, tumour stage, tumour diameter, tumour histology, lymphovascular space involvement (LVSI), recurrence status, recurrence date, primary treatment modality, the surgery type, and date of death from patients' records. Although the factors of survival in cervical cancer are well known, they are not obvious in the neuroendocrine subtype.

## 2. Materials and Methods

Records of 453 patients with cervical cancer who were admitted to our clinic between March 2004 and March 2021 were reviewed, but only patients whose cervical biopsy reports indicated neuroendocrine cancer were included in the study. This retrospective study includes 9 cases diagnosed with neuroendocrine cell cancer of the cervix between March 2004 and March 2021 in Akdeniz University Gynecological Oncology Outpatient Clinic. Patients were diagnosed based on examination and reporting of the biopsy samples collected by the academic staff of the pathology department. The data were collected from patients who consented to participate in the study (consent was taken from the first-degree relatives of the deceased ones) by utilizing an electronic data system, while the whole process was monitored and approved by the Hospital Ethics Committee.

Patients with cervical cancer with other histological types of the cervix, those who did not participate in regular follow-up, and those with metastatic neuroendocrine cancer of the cervix were excluded from the study. Age, initial diagnosis date, body mass index, smoking, menopause status, HPV status and type, family history, immunohistochemical staining patterns, gravida, parity, abortion, accompanying disease, tumour stage, tumour diameter, tumour histology, LVSI, presence of recurrence, recurrence date, primary treatment modality, the surgery type, and survival were obtained retrospectively from the electronic data system. Follow-up of the patients was performed every 3 months for the first 2 years and every 6 months in the following years in order to detect recurrence as soon as possible. Symptoms of the patients were evaluated, and pelvic examinations were performed at each visit. Periodic imaging methods such as chest X-ray, pelvic ultrasound, and computed tomography (CT) or positron emission tomography-computed tomography (PET-CT) were used to detect relapse in the interim period until relapse. Overall survival (OS) was calculated as the time from the initial diagnosis to death. Progression-free survival (PFS) was calculated as the time after treatment until the first recurrence was detected.

For descriptive statistics, the mean, standard deviation, median, Min–Max values, and frequencies were used, considering whether there was a normal distribution or not. The categorical data were expressed in numbers and percentages (%). Kaplan–Meier survival analysis was used for progression-free survival (PFS) and overall survival (OS). “The Statistical Package for the Social Sciences (SPSS) 23” program was used in the analysis of the data.

## 3. Results

**Figure 1:**
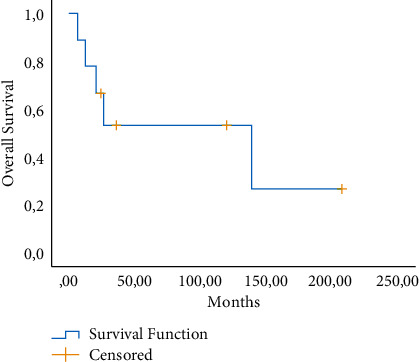
Kaplan–Meier overall survival (OS) rate for all patients.

**Figure 2:**
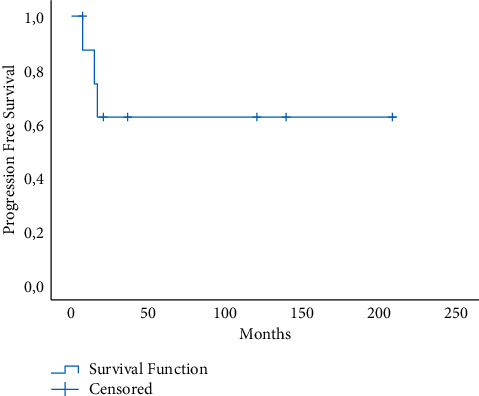
Kaplan–Meier progression-free survival (PFS) rate of all patients.

The demographic characteristics of the patients are shown in Table 1. We analyzed 9 patients (1.98%) with neuroendocrine subtypes out of 453 patients diagnosed with cervical cancer. On histopathological examination, 8 tumours were classified as small cell carcinoma and 1 tumour was classified as large cell neuroendocrine cancer. The ages of the patients ranged from 33 to 78 years, with an average age of 58.5 years, and 5 of them were illiterate. According to the International Federation of Gynecology and Obstetrics' (FIGO) clinical-stage definitions, 3 cases were early stage, 5 cases were locally advanced, and 1 case was metastatic. The tumour size ranged from 2.5 to 12 cm, with an average of 3.6 cm. In the gynecological examination, 4 patients had an ulcerated tumour, 3 patients had an infiltrative character, and 2 patients had a vegetative appearance. 3 patients were at stage 1b2 at the time of diagnosis, 4 patients were at stage 2b, 1 patient was at stage 3c2r, and 1 patient was at stage 4b when the stage subgroups were analyzed. The further detailed analysis showed 6 cases had deep stromal invasion, 7 cases had lymph vascular invasion, and 3 cases had lymph node involvement. In immunohistochemical staining, neuroendocrine markers including synaptophysin (SYN) in 5 patients, CD56 (N-CAM) in 3 patients, neuron-specific enolase (NSE) in 3 patients, S-100 protein in 1 patient, and chromogranin (CHG) in 5 patients were observed. While HPV was negative in 2 patients, it was not known the remaining 7 patients' HPV status. While 3 patients reported insufficient smear results, 2 patients were malignancy negative, 2 patients' reports were HSIL, and 2 patients had never had a smear test until the time of the diagnosis. Regarding the surgical treatment methods of the patients, the primary treatment modality of 6 patients was surgery and adjuvant chemoradiotherapy (CRT), 3 patients received primary CRT. 5 patients underwent type 3 radical hysterectomy + bilateral salpingoophorectomy and bilateral pelvic paraaortic lymphadenectomy, 1 patient underwent type 3 radical hysterectomy + bilateral salpingoophorectomy and bilateral pelvic lymphadenectomy, laparoscopic pelvic paraaortic lymphadenectomy was performed in 1 patient, and 2 patients received medical treatment. Clinical characteristics of 9 patients with cervical neuroendocrine tumour are shown in Table 2. The main chemotherapeutic agents used in our patients were cisplatin/carboplatin and etoposide. Multiple chemotherapeutic agents (cisplatin + etoposide and cyclophosphamide + etoposide) were used on only one of our patients due to her advanced stage. The mean follow-up period of the patients was 26 months (ranging from 6 to 208). Recurrence was found in 3 cases, while 5 cases resulted in death. One of these patients had a recurrence in the vaginal cuff, and the other had a recurrence in the pelvic lymph node. The 5-year survival rate was 53.3%, demonstrated in Figure 1. The 5-year PFS was 62.5% and is presented in Figure 2. Since paraneoplastic syndrome was not found in any of our patients, further investigation was not required.

## 4. Discussion

Neuroendocrine carcinoma of the cervix accounts for less than 5% of all cervical cancers [11]. Our study presents that the incidence of neuroendocrine cancer (1.98%) is below 2% similar to previous studies. Since they have a more aggressive course and worse prognosis compared to the more common histological subtypes of cervix cancer (squamous cell cancer and adenocarcinoma), it should be investigated whether they metastasize at the time of diagnosis. Because of its low incidence, standard treatment has not yet been determined. Therefore, the treatment scheme and factors affecting survival have not been clearly revealed. However, age, tumour size, lymphovascular area involvement, lymph node metastasis, stromal invasion depth, parametrial involvement, treatment modality [12–19], and FIGO stage [20] have been discussed in the literature.

Although the factors of survival in cervical cancer are well known, they are not obvious in the neuroendocrine subtype. We calculated the 5-year survival rate as 53.3% which is significantly higher than the evidence of previous studies [21–23]. In our study, the average overall survival of the patients was 26 months. Based on the 3 patients who suffered relapses, it was deduced that relapses generally occur between the first and second years while deaths usually happen in the first 2 years. Therefore, one should be cautious in terms of metastasis, especially between the 1st and 2nd years in the follow-up period. However, in a meta-analysis by Xu et al., it was stated that this attention should be paid between the 2nd and 3rd years [21].

In our study, the most common subtype of neuroendocrine cancer was small cell cancer, and immunohistochemical staining patterns were consistent with the literature. Even if some small cell neuroendocrine cancers were negatively stained immunohistochemically in terms of neuroendocrine markers [2], all of our patients were positively stained by at least two markers according to the diagnostic criteria. Neuroendocrine tumours do not have any clinical features to distinguish them from other histological types of cervical cancer, and they have similar clinical symptoms to those of cervical cancer. In our study, the examination findings of the cervix (vegetative, infiltrative, or ulcerative appearance) and the main complaints of the patients (postcoital bleeding, vaginal discharge, irregular bleeding, and pelvic pain) were not characteristic. HPV, which is involved in the aetiology of cervical cancer, has also been observed in neuroendocrine tumours, even as in squamous cell cancer, it has been pointed out as the main factor responsible for cancer development [4, 5]. In our study, only 2 of 9 patients had a negative HPV test, while the other 7 patients did not have any HPV test due to the fact that HPV screening has just recently been integrated into Turkey's medical infrastructure. Only 1 patient was *p*16 positive, indicating the presence of high-risk HPV in the aetiology. Positive detection of HPV can help distinguish cervical neuroendocrine tumours from endometrial neuroendocrine tumours [3]. Surgery and adjuvant CRT, which are the accepted treatment methods for other histological types of cervix cancer, were applied to our patients with early stages, and the most used chemotherapeutic agents were etoposide and cisplatin/carboplatin. It has been reported that different chemotherapeutic combinations have been used in cases with local progression or relapse [2, 24]. However, a standard treatment regimen has not yet been defined due to the low incidence of this neoplasia, the lack of prospective studies, and well-organized studies in the literature. In addition to these, the treatments we administer to the patients are generally based on the treatment schemes administered in neuroendocrine tumours located outside the cervix, since neuroendocrine tumours mostly involve the gastrointestinal tract and lung rather than the cervix in humans [7].

One of the reasons for the poor prognosis of our patients is that all of our patients had poorly differentiated subtypes of neuroendocrine neoplasia. Besides, well-differentiated neuroendocrine tumours such as typical carcinoma and atypical carcinoma are rarely seen in the cervix [24]. Another reason for the poor prognosis is that neuroendocrine tumours invade more lymphovascular space and lymph nodes compared to squamous cell cancer and adenocarcinoma at the time of diagnosis. Therefore, the 5-year and disease-free survival rates are lower, with an average of approximately 34% [2]. In our study, 5-year survival was calculated higher (53.3%) compared to the literature. This may be due to the low number of our patients.

The limitations of our study are that it was a retrospective study, there are no prospective randomized controlled studies, and we could not find prognostic factors since the number of our population is low.

## 5. Conclusion

Neuroendocrine cell tumour is a rare subtype of the cervix with a poor prognosis since it progresses aggressively and invades deeply. While radical surgery and adjuvant CRT are used for early-stage patients, primary chemoradiotherapy can be used on more advanced stages. Unfortunately, there is yet no standard and first-line treatment protocol due to the limited number of comparative studies of surgery, chemotherapy, and radiotherapy-based treatment schemes. Despite aggressive treatment, most patients develop metastasis; therefore, new treatment schemes should be defined. Further research is required to determine such scheme.

## Figures and Tables

**Table 1:** Patient characteristics (*N* = 9).

		Number/mean/median	%/SD/Min–Max
Age (mean, SD)		58, 5	±16, 1
Gravida (median, Min–Max)		5	2–8
Parity (median, Min–Max)		4	1–7
BMI (mean, SD)		24, 4	±2, 3
Ca 125 (median, Min–Max)		24, 5	8, 1–264, 2
Cigarette (mean, SD)	Pack/year	12, 1	±9, 5
Mode of delivery	NVD	7	22, 2
	C/S	2	77, 8
Education status	Illiterate	5	55, 6
	Primary school ≤	4	44, 4
Smear result	Absent	5	55, 6
	Normal	2	22, 2
	HSIL	2	22, 2
First complaint	Postcoital bleeding	3	33, 3
	Pelvic pain	2	22, 2
	Postmenopausal bleeding	3	33, 3
	Vaginal discharge	1	11, 1
Histological type	Neuroendocrine	9	1, 98
	Others	444	98, 01
Stage	Early	3	33, 1
	Locally advance	5	55, 5
	Metastatic	1	11, 1
Stage subgroup (FIGO)	1b2	3	33, 3
	2b	4	44, 4
	3c2r	1	11, 1
	4b	1	11, 1
Treatment modality	Primary CRT	3	33, 1
	Surgery + adj. CRT	6	66, 6
L/S	Done	2	22, 2
	Not done	7	77, 8
LVSI status	Negative	2	22, 2
	Positive	7	77, 8
Lymph status	Negative	6	66, 7
	Positive	3	33, 3
Recurrence	Present	3	33, 3
	Absent	6	66, 7
Death	Present	5	55, 6
	Absent	4	44, 4
Follow-up (month)		26 (6–208)	

FIGO, International Federation of Gynecology and Obstetrics; BMI, body mass index; NVD, normal vaginal delivery; CS, caesarean section; CRT, chemoradiotherapy treatment; and LVSI, lymphovascular space involvement.

**Table 2:** Clinical characteristics of cervical neuroendocrine tumours.

Patient name	Age	Stage	Surgical procedure by	Type of surgery	Tumour diameter (cm)	Recurrence status	Preoperative CRT (C + E)	Postoperative CRT (C + E)	PFS (mo)	OS (mo)	Status
Ü.S.	65	2b	L/T	Type III RH + BSO + BPPLND	12	+		+	7	12	Dead
Z.A.	70	2b	L/T	Type III RH + BSO + BPPLND	5	−		+	20	20	Dead
T.Ö.	33	1b2	L/T	Type III RH + BSO + BPPLND	3	−		+	208	208	Alive
M.U.	78	1b2	L/T	Type III RH + BSO + BPLND	2, 5	−		+	139	139	Dead
Y.A.	78	2b	—	—		+	+		14	26	Dead
Ş.Ç.	39	3c2r	L/S	Lymphadenectomy		−	+		120	120	Alive
A.D.	48	1b2	L/S	Type III RH + BSO + BPPLND	3, 2	−		+	36	36	Alive
E.K.	58	2b	L/T	Type III RH + BSO + BPPLD	10	+		+	16	24	Alive
M.F.A	58	4b	—	—		−	+		6	6	Dead

L/T: laparotomy; L/S: laparoscopy; RH + BSO + BPPLND: radical hysterectomy + bilateral salpingoophorectomy + bilateral pelvic paraaortic lymphadenectomy; mo: month; C + E: cisplatin + etoposide.

## Data Availability

The data used to support the findings of this study are available from the corresponding author upon reasonable request.

## Supplementary Materials

Supplementary Materials STROBE Statement: checklist of items that should be included in reports of cross-sectional studies.
